# Nanomaterials as Redox Mediators in Laccase-Based Amperometric Biosensors for Catechol Assay

**DOI:** 10.3390/bios12090741

**Published:** 2022-09-08

**Authors:** Olha Demkiv, Galina Gayda, Nataliya Stasyuk, Olena Brahinetz, Mykhailo Gonchar, Marina Nisnevitch

**Affiliations:** 1 Institute of Cell Biology National Academy of Sciences of Ukraine, 14/16, Dragomanova Str., 79005 Lviv, Ukraine; 2 State Institution Institute of Blood Pathology and Transfusion Medicine National Academy of Medical Sciences of Ukraine, 45, General Chuprinka Str., 79044 Lviv, Ukraine; 3 Department of Chemical Engineering, Ariel University, Kyriat-ha-Mada, Ariel 4070000, Israel

**Keywords:** laccase, electroactive nanoparticles, amperometric biosensor, catechol analysis

## Abstract

Laccase is a copper-containing enzyme that does not require hydrogen peroxide as a co-substrate or additional cofactors for an enzymatic reaction. Nanomaterials of various chemical structures are usually applied to the construction of enzyme-based biosensors. Metals, metal oxides, semiconductors, and composite NPs perform various functions in electrochemical transformation schemes as a platform for the enzyme immobilization, a mediator of an electron transfer, and a signal amplifier. We describe here the development of amperometric biosensors (ABSs) based on laccase and redox-active micro/nanoparticles (hereafter—NPs), which were immobilized on a graphite electrode (GE). For this purpose, we isolated a highly purified enzyme from the fungus *Trametes zonatus*, and then synthesized bi- and trimetallic NPs of noble and transition metals, as well as hexacyanoferrates (HCF) of noble metals; these were layered onto the surfaces of GEs. The electroactivity of many of the NPs immobilized on the GEs was characterized by cyclic voltammetry (CV) experiments. The most effective mediators of electron transfer were selected as the platform for the development of laccase-based ABSs. As a result, a number of catechol-sensitive ABSs were constructed and characterized. The laccase/CuCo/GE was demonstrated to possess the highest sensitivity to catechol (4523 A·M^−1^·m^−2^) among the tested ABSs. The proposed ABSs may be promising for the analysis of phenolic derivatives in real samples of drinking water, wastewater, and food products.

## 1. Introduction

Laccase (EC 1.10.3.2, *p*-diphenol:oxygen oxidoreductase) belongs to the family of multicopper blue oxidase, which is typically found in plants and fungi. Laccase reduces molecular dioxygen to water while catalyzing the oxidation of a variety of compounds including polyamines, lignins, aryldiamines, a number of inorganic ions, and phenol derivatives [1,2].

Phenolic compounds are important markers in medicine, pharmaceuticals, the food industry, and pollution control. Laccase-based monitoring of phenol derivatives is a promising analytical approach to improve food production and environmental safety [3,4,5].

Laccase is generally used in different industries, including food, textile, wood, pharmaceutical industries, and others [4,5,6,7,8,9]. The global laccase market is projected to reach USD 3 million in 2022 and is forecasted to reach USD 4 million by 2028 (adjusted for a compound annual growth rate of 4.3% during the review period). Among those applications, the textile industry is the largest field [10]. Additionally, laccases are very useful in green organic syntheses, due to their efficient catalyzing polymerization of phenolic and aromatic compounds. This process was seen to be initiated by the formation of a radical cation, followed by intermolecular attacks to produce dimmers/oligomers/polymers, which are useful in the food, wood, medical, and cosmetic industries [1,2,3,7,8,9,10,11]. 

Currently, there are six major laccase suppliers in the world: Novozymes, DuPont, Amano Enzyme, Yiduoli, Sunson, and Denykem; together they comprise 98% of the total market [10]. Novozymes is the global leader, with a production share of 66%. DuPont is the second largest producer. Since market concentration in this industry is high, the search continues for new cost-effective producers of laccase and the development of simple technologies for its isolation [1,2,3,4,12,13,14,15]. The fabrication of sensitive analytical methods based on laccase also remains relevant [5,12].

The growing discoveries of novel varieties of laccase have demonstrated that laccase classification and investigation are not so simple [11,12,13,14,15,16]. The classification of multi-copper oxidases assigned as laccases remains a challenge too [1,2,3,9,11]. Because of the great variety among the known laccases, for the sake of simplicity, Reiss et al. [2] proposed the term “laccase-like multi-copper oxidase” (LMCO) to distinguish them from the laccase enzyme originally identified from the sap of the lacquer tree *Rhus vernicifera* [17].

In our previous work, the screening of fungi and mushroom cultures for their capability to synthesize laccase was carried out, the best producers of laccase were selected, the optimal conditions were estimated for cell cultivation to achieve the highest productivity of these enzymes, and novel laccase-based amperometric biosensors (ABSs) were proposed [18,19]. 

Among many recent advanced detection platforms, laccase-based biosensors are superior for their rapid, effective, and online use by in situ devices for the determination of phenolic compounds [19,20,21,22,23]. In enzyme-based biosensors, immobilization methods play a significant role. Future potential has been proposed for novel achievements in laccase immobilization techniques (entrapment, adsorption, cross-linking, and covalent) on new nanocomposites, including electroactive nanomaterials for laccase-based biosensors [19,23].

Numerous nanostructured carriers have been developed to advance traditional enzyme immobilization strategies, including carbon nanotubes, graphene and its derivatives, nanoparticles, nanoflowers, and metal-organic frameworks with unique properties. These platforms for enzyme immobilization have a high surface-to-volume ratio, high surface area, robust chemical and mechanical stability, surface pendant functional groups, and outstanding electrical characteristics, resulting in a higher concentration and stabilization of the immobilized enzyme [3,12,19,20,21].

NPs of various chemical structures and materials are known to be useful in the development of electrochemical sensors. Metal, metal-oxide, semiconductor, and composite NPs perform various functions in electrochemical transformation schemes. Their mediator properties are the most important factors [22,23,24].

The growing need in the catalytic industry can be met by multi-enzymes co-immobilized on these nanostructured materials that form the support matrices. Precise coordination between the target enzyme molecules and the surface pendant multifunctional nanostructured carriers has led to an effective and significant contribution in various novel applications for industry, biotechnology, and biomedicine [22,23].

The aim of the current work was to develop ABSs for catechol assay. Catechol (1,2-benzenediol) is a natural phenolic compound that is widely used in a variety of industrial applications including production of plastic, rubber, pesticides, and pharmaceuticals. In nature, catechol was detected in higher plants, including fruits, vegetables, tobacco, tea, and other products. As a toxic compound, catechol even at low concentrations (in foods and cigarette smoke) may cause mutagenic and cancerogenic transformations in humans, including renal tube degeneration and decreases in liver functions [25]. As catechol has been found in a variety of organisms as well as in the environment, various institutions such as healthcare, environmental safety, and industrial quality control need reliable, reproducible analytical methods to monitor toxic catechol [25,26].

Many techniques have been proposed for the detection of catechol and other derivatives of phenol, including high-performance liquid chromatography, thin-layer chromatography, UV-Vis spectrophotometry, IR spectrometry, fluorescence, surface plasmon resonance, and others [23,27,28,29]. Being complicated and time consuming, these methods require highly skilled manpower and are difficult to use for routine analyses. Sensitive, rapid, and simple electrochemical analytical methods—namely (bio)sensors, especially with the usage of electroactive 3D nanomaterials—are promising alternatives for traditional methods of catechol assay [23,30,31,32,33,34,35]. 

Fabrication of amperometric (bio)sensors for fast, sensitive, and, at the same time, selective assay of catechol (or any other individual substrate of laccase) is a serious challenge for electrochemists today. Many reports concerning specific catechol detection and describing laccase-based biosensors [28,36,37,38] as well as non-enzymatic sensors have been published [27,33,34,35,39,40,41,42]. All the reported methods are based on using advanced nanomaterials. It was demonstrated that selectivity of laccase-based methods depended on the producer of the enzyme [2,9,11,12,20], the mode of enzyme immobilization/fixation [23,28], the nature of nanomaterials and composition of a reaction mixture [31,36,37,38], working potential [39], presence of organic solvents [43], and many other parameters.

We report here on the development of the ABSs for catechol assay, based on purified fungal laccase which was co-immobilized with synthesized redox-active NPs on graphite electrodes. We characterize the analytical properties of the obtained ABSs and demonstrate the applicability of the most sensitive ABS for the assay of catechol phenolic derivatives in a real sample of wastewater.

## 2. Materials and Methods

### 2.1. Reagents and Enzyme

Copper(II) sulphate, hydrogen tetrachloroaurate(III) trihydrate, cerium(III) chloride, nickel(II) sulfate, chloroplatinic(IV) acid, palladium(III) chloride, *o*-dianisidine, 2,2′-azinobis (3-ethylbenzothiazoline-6-sulfonate) diammonium salt (ABTS), catechol, and other reagents and solvents used in this work were purchased from Sigma-Aldrich (Steinheim, Germany). All reagents were of analytical grade and were used without additional purification. All solutions were prepared using ultrapure water. Laccase from *Trametes zonatus* was purified and characterized as described by us earlier [18,19].

### 2.2. Synthesis of NPs 

Chemical synthesis of hexacyanoferrates (HCFs) was performed by mixing equal volumes of 50 mM K_4_Fe(CN)_6_ solution with 50 mM solutions of PdCl_3_, AgNO_3_, and AuCl_3_. 

“Green” NPs, namely gCu(II)HCFs and gFe(III)HCFs, were synthesized using enzyme flavocytochrome *b_2_* [44]; gAu was obtained using extracellular metabolites of yeast [45]. 

Bi-and trimetallic NPs were synthesized by the chemical reduction of metal ions from appropriate salts [46,47] or by the chemical bath deposition method [48]. PtRu/NPs, NiPtPd/NPs, and CuCo/NPs (further—PtRu, NiPtPd, and CuCo, respectively) were obtained and characterized as described in our recent works ([46,47,48], respectively). 

All the synthesized NPs were collected by centrifugation, washed with water, and stored as a water suspension at 4 °C until use [48]. 

### 2.3. Apparatus and Statistical Analysis 

The amperometric biosensors (ABSs) were evaluated using constant-potential amperometry as described in detail in our previous work [35]. The amperometric experiments were carried out in triplicate, using a graphite rod (3.05 mm diameter) as a working electrode, a Pt-wire as a counter electrode, and an Ag/AgCl/KCl (3 M) reference electrode, which were placed together in a glass electrochemical cell with a working volume of 20 mL. The analytical parameters of the electrodes were estimated and statistically processed as described earlier [44,45,46,47]. Morphological analyses of the NPs by scanning electron microscopy (SEM) were performed as described earlier [44,45,46,47,48].

### 2.4. Characterization of the NPs for Their Redox Electroactivity 

The electrochemical properties of the synthesized NPs on the graphite electrodes (GEs) were studied by cyclic voltammetry (CV) using a solution of 10 mM K_3_FeCN_6_ with 0.1 M KCl in the range from −800 to +800 mV with a scan rate of 50 mV·min^−1^ at room temperature. 

For the GE modification, a 0.01 mL aliquot of NPs (0.1 mg/mL) was deposited onto the GE surface and air-dried at room temperature. The NPs/GE was covered with 5 μL of 0.5% neutralized Nafion solution, dried, washed with a 50 mM sodium acetate buffer of pH 4.5 (further—NaOAc buffer), and stored at 4 °C until used. 

### 2.5. Fabrication and Characterization of the Laccase/NP-Modified Graphite Electrode 

To develop the laccase and NP-based electrode, 5 μL solution of laccase with an activity of 20 U mL^−1^ was dropped onto the surface of the NPs/GE and air-dried. The resulting laccase/NPs/GE was covered with Nafion, dried, washed, and stored as described in Section 2.4. The most effective laccase/NPs/GE was studied in more detail as an ABS for determination of catechol. In the selectivity tests, two methods of fixation of laccase/NPs on the surface of GE were compared: one using Nafion film, and the other using dialysis tubing cellulose membrane (DM, D9777-100FT) from Sigma Aldrich (Austin, TX, USA). 

### 2.6. Application of the Laccase/CuCo-Based ABS for Catechol Assay in the Model and Real Samples

The samples were tested using the graphical calibration method in a variant of the standard addition test (SAT). This approach is usually applied to avoid the possible interference effects of the accompanying components of analyte; it is based on multiple additions of the standard. The principle of the SAT method and the algorithm for the calculation of an analyte’s concentration (C) were described in detail in our previous work [46]. C in the tested sample is calculated from the equation C = A·N/B, where A and B are linear regression parameters and N is the dilution coefficient. Values of A,B, N, and C are presented in correspondent calibration graphs.

The sample of wastewater was obtained from the factory “Dezomark”, which produces the disinfectant substances and detergents (Lviv, Ukraine). Extract of the green tea “Greenfield” (Greenfield Incorporation LLC, Kyiv, Ukraine) was prepared by incubation of 2.5 g dry leaves in 50 mL water (35 °C) for 20 min under shaking [34]. Both assays were performed in *triplicate* for two dilutions of the wastewater and for three dilutions of the tea extract.

## 3. Results

### 3.1. Selection of the Optimal Redox Nanomediators 

For improvement of electron exchange between the surface of the working electrode and the enzyme, NPs with high electro-mediator activity may be used. To obtain such compounds, NPs of noble and transition metals, as well as metal-based composite materials, were synthesized using chemical and biological methods for metal ion reduction. 

To select the best mediators of electron transfer, the synthesized NPs were screened for their electroactivity using CV. Redox properties of the control GE and the NPs/GE were tested with a scan rate of 50 mV·min^−1^, in NaOAc buffer, at 23 °C. Such conditions were experimentally chosen in our previous works [24,44] as being optimal for characterization of metallic NPS. That is why the subsequent experiments in this study were carried out under these conditions.

**Figure 1:**
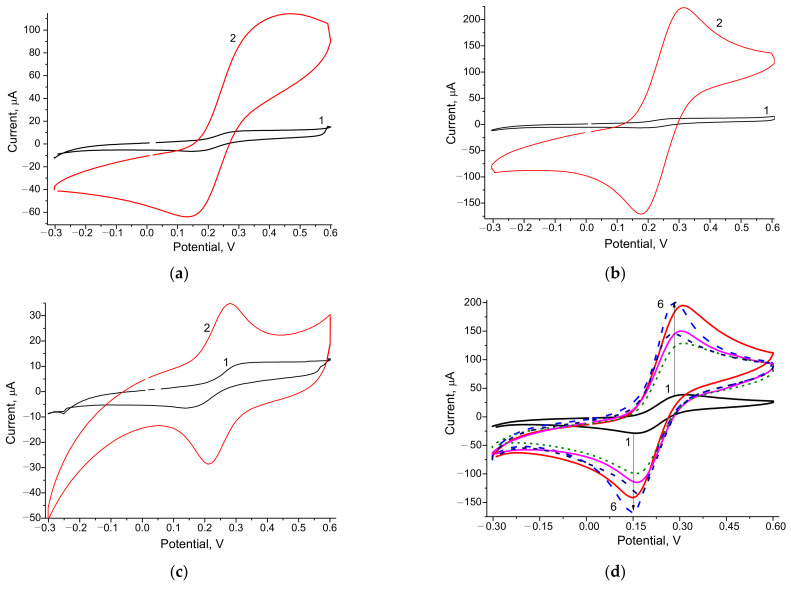
CV profiles as outputs from increasing concentrations of K_3_Fe(CN)_6_ of the control electrode GE (1—**a**–**d**) and the modified electrodes: PdHCF/GE (**a**, 2); CuCo/GE (**b**, 2); NiPtPd/GE (**c**, 2); PtCeHCF/GE (**d**, 2), AuCoHCF/GE (**d**, 3), AuHCF/GE (**d**, 4), PtHCF/GE (**d**, 5), AgHCF/GE (**d**, 6).

Numerous NP-modified electrodes were screened for their ability to reduce K_3_Fe(CN)_6_ to K_4_Fe(CN)_6_. The electrochemical properties of the electrodes were studied by CV. The CV profiles of current signals of different NP/GEs represented outputs from increasing concentrations of K_3_Fe(CN)_6_, shown in Figure 1. 

According to the results of the CV study, all tested NPs were electroactive, since the peaks of oxidation and recovery of the NP/GEs were substantially higher than those of the control (unmodified) GE. It is worthwhile to emphasize that determining the value of the optimal working potential for achieving the maximal current difference between the GE and the NPs/GE was essential for subsequent experiments. The lower optimal potential is necessary to avoid the effect of possible interfering substances on the electrode as a response to the presence of oxygen. This requirement is relevant for the construction of ABSs and their exploitation for the analysis of real samples (food products, biological liquids, and others). 

As shown in Figure 1, the studied NPs showed significantly increased efficiency of electron transfer, making them promising electroactive mediators for biosensors.

### 3.2. Development of Catechol-Sensitive Laccase-Based Amperometric Nanobiosensors 

Laccase is a copper-containing enzyme that does not require hydrogen peroxide as a co-substrate or additional cofactors for an enzymatic reaction. Nanomaterials, which are usually exploited for biosensor construction, can carry out several functions as platforms for enzyme immobilization, mediators, and signal amplifiers. Direct immobilization of the enzyme on the surface of the electrode can lead to its partial denaturation, whereas on the surface of the NPs, their bioactivity can be better conserved due to complexation.

**Figure 2:**
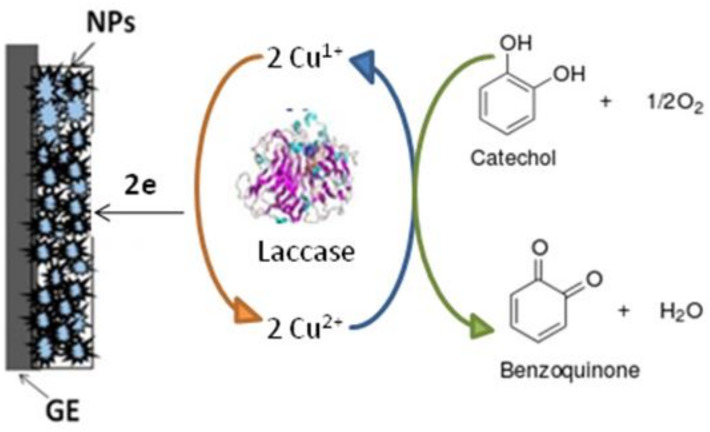
The principal scheme of catechol determination using a laccase-based ABS.

The principle of detection with the constructed ABSs is presumably based on the reduction of oxidized electroactive products, which are formed as a result of laccase-mediated catalysis. In the case of catechol degradation, the product formed for further reduction is semiquinone. The scheme of electron transfer during conversion of catechol as a substrate of the laccase in the ABS is presented in Figure 2. Coupled with laccase, the most effective electrochemically active redox mediators were used. 

**Figure 3:**
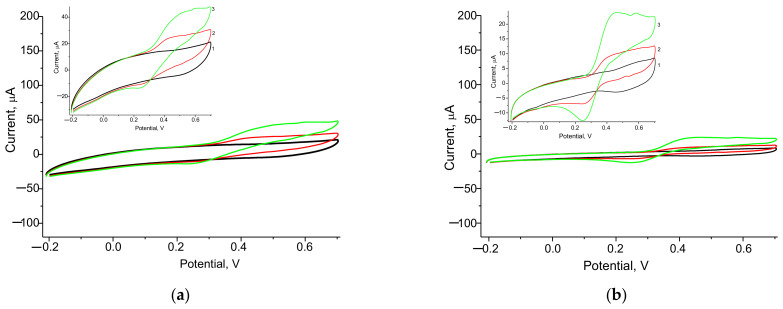

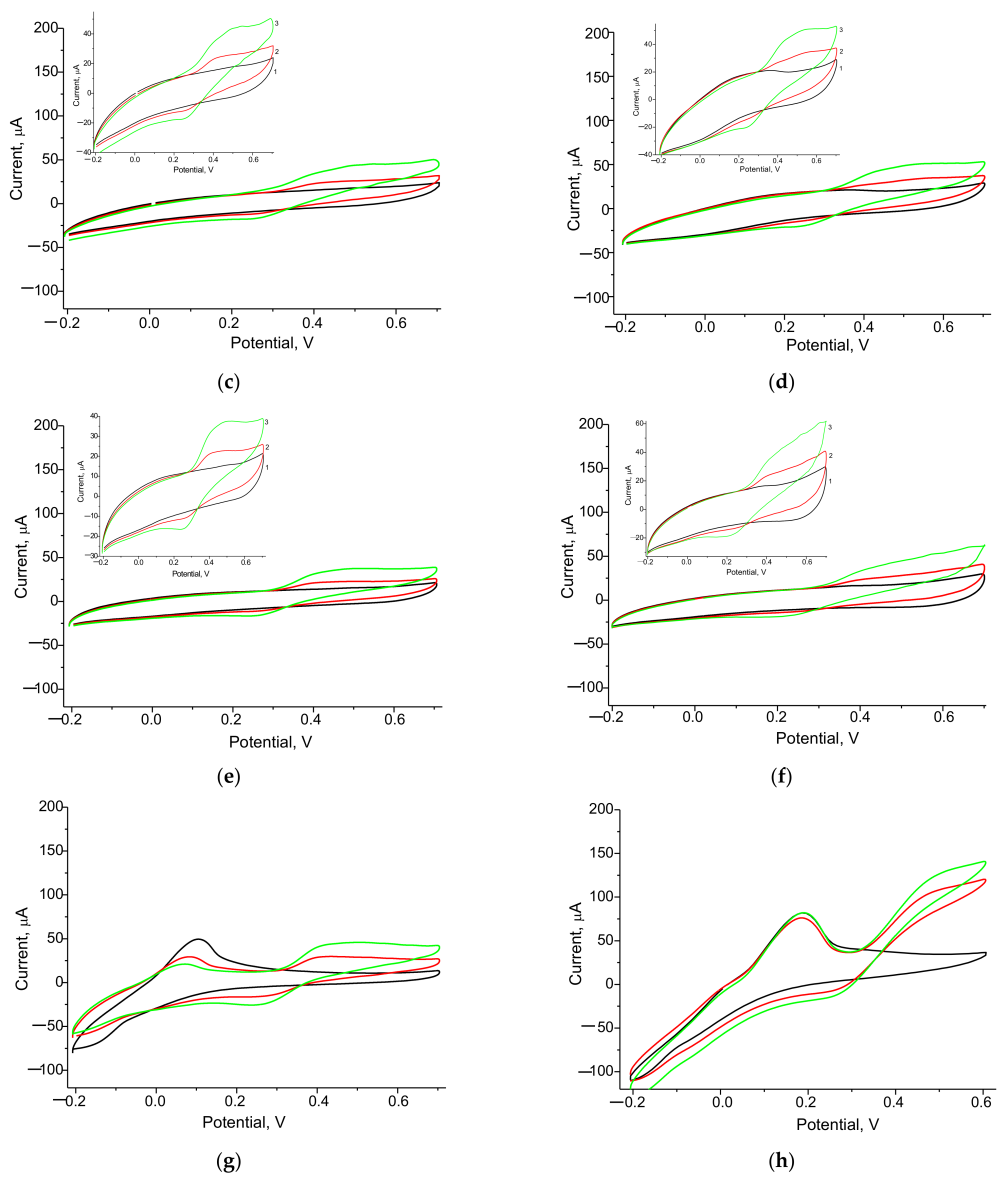
Cyclic voltammograms for the constructed ABSs, as current responses upon addition of catechol in varying concentrations: 0 mM (1, black), 0.25 mM (2, red); 0.50 mM (3, green). ABSs: laccase/GE (**a**), laccase/PdHCF/GE (**b**), laccase/PtHCF/GE (**c**), laccase/AuHCF/GE (**d**), laccase/AgHCF/GE (**e**), laccase/PtCeHCF/GE (**f**), laccase/NiPtPd/GE (**g**), and laccase/CuCo/GE (**h**).

To select an appropriate working potential for a laccase-based ABS, CV was used. The CV profiles of the constructed ABSs, under the addition of catechol as a substrate of laccase, were compared with CV profiles without the analyte. As shown in Figure 3, an increase in oxidation and reduction peaks indicated an increase in the efficiency of electron transfer between the electroactive product of the laccase reaction and the modified GE surface, which confirms the redox mediator activity of the NPs. 

It is clear from the CV results for laccase/GE (Figure 3a) that the peak of reduction, as an output upon catechol addition, appeared in the range 200–250 mV. Thus, for subsequent experiments, the working potential 230 mV was selected as optimal. 

### 3.3. Properties of the Developed Laccase-Based ABS

#### 3.3.1. Analytical Characteristics 

**Figure 4:**
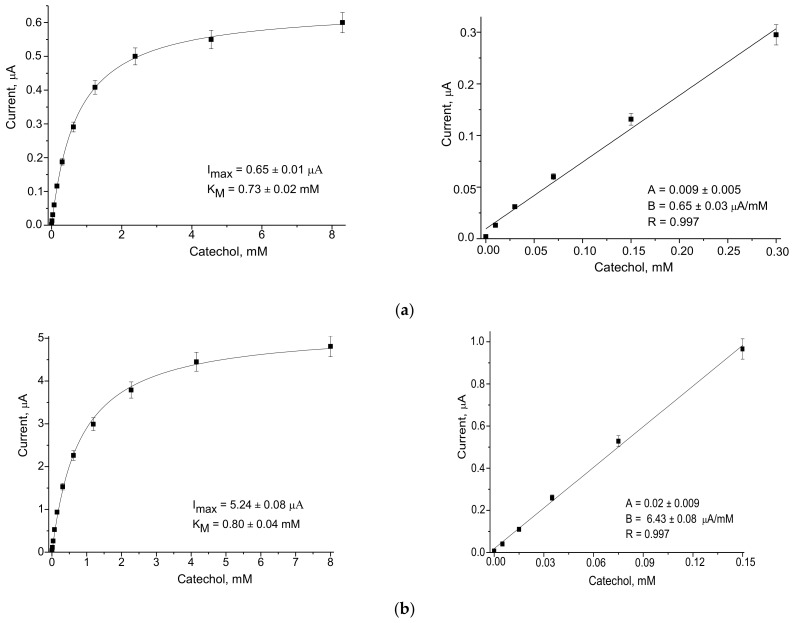

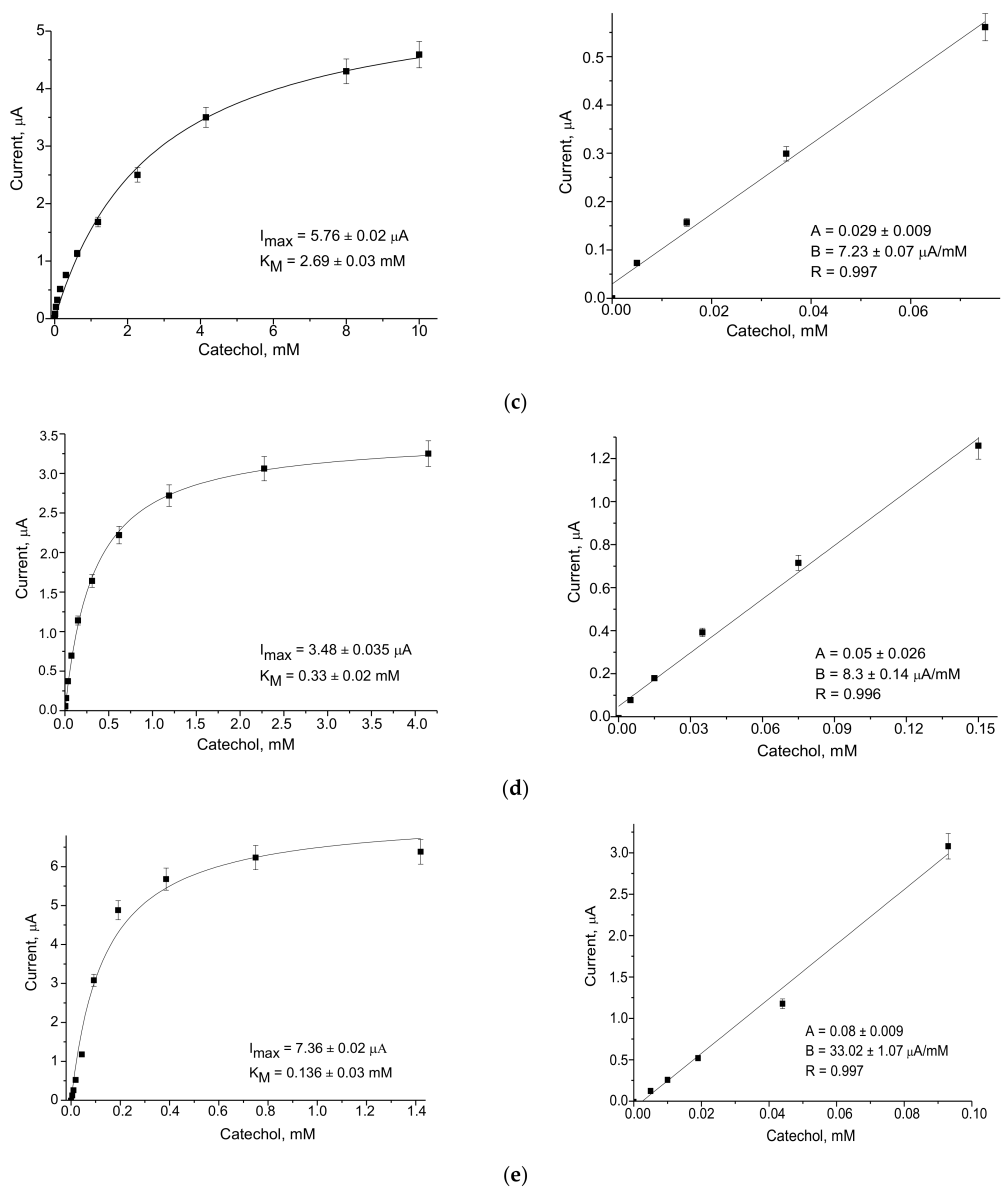
Calibration curves for catechol determination in wide (left) and linear (right) ranges using the highly sensitive ABSs: (**a**) control electrode laccase/GE without NPs; (**b**) laccase/AgHCF/GE, (**c**) laccase/NiPtPd/GE, (**d**) laccase/PdHCF/GE, (**e**) laccase/CuCo/GE. Abbreviations: B—slope of the calibration graph; R—the correlation coefficient of linear regression.

**Figure A1:**
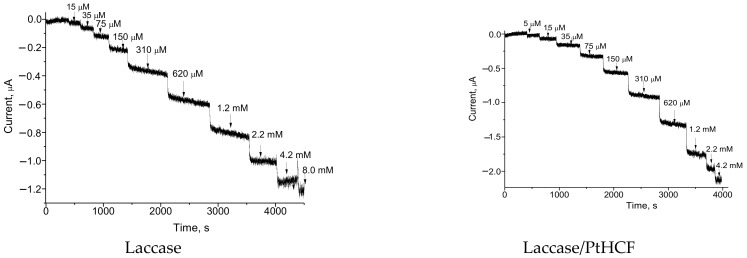

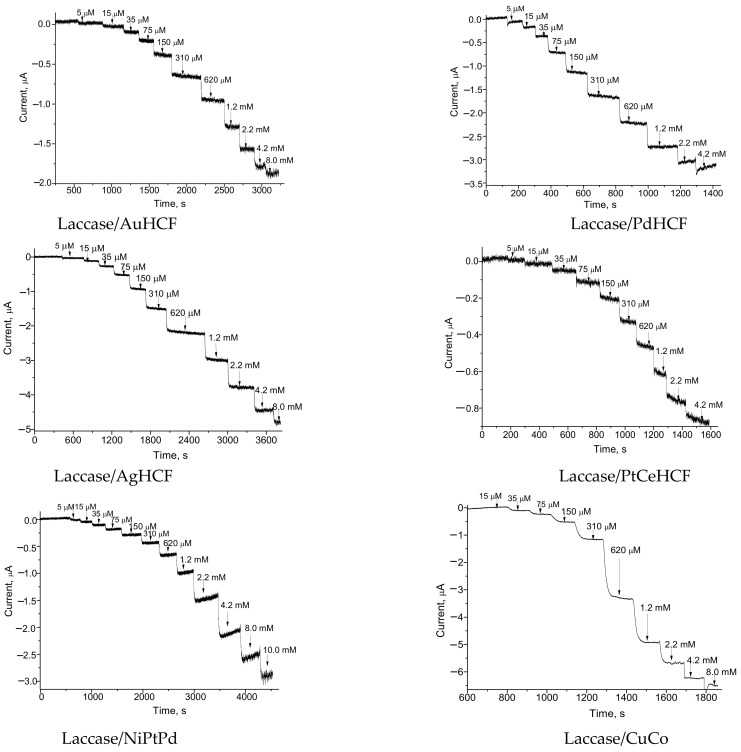
Chronoamperograms of the current responses to catechol addition from the laccase/NP-based ABSs. Conditions: Ag/AgCl (reference electrode) in NaOAc buffer. Working potential: +230 mV.

**Figure A2:**
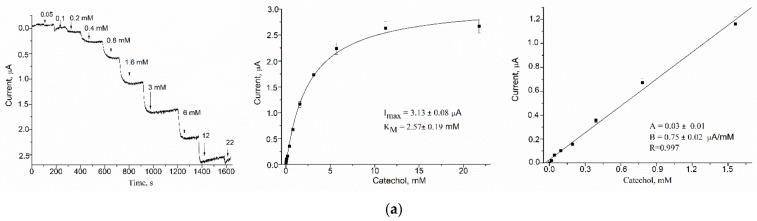

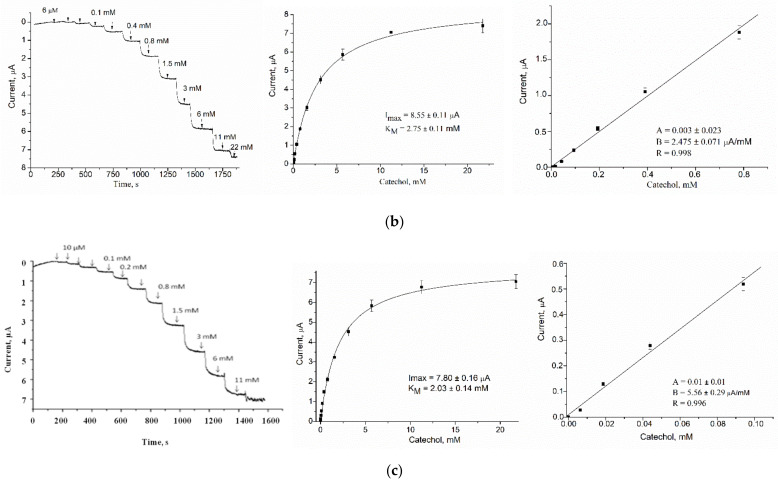
Chronoamperograms (left) and calibration curves for catechol determination in wide range (middle) and linear range (right) for the ABSs: laccase/GE (**a**), laccase/gFeHCF/GE (**b**), and laccase /gCuHCF/GE (**c**). Conditions: Ag/AgCl (reference electrode) in NaOAc buffer. Working potential: +230 mV.

**Figure A3:**
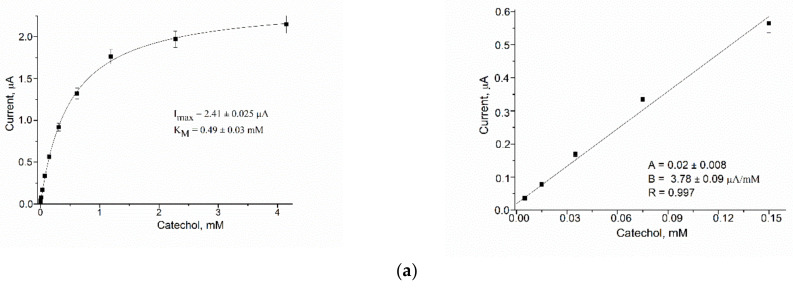

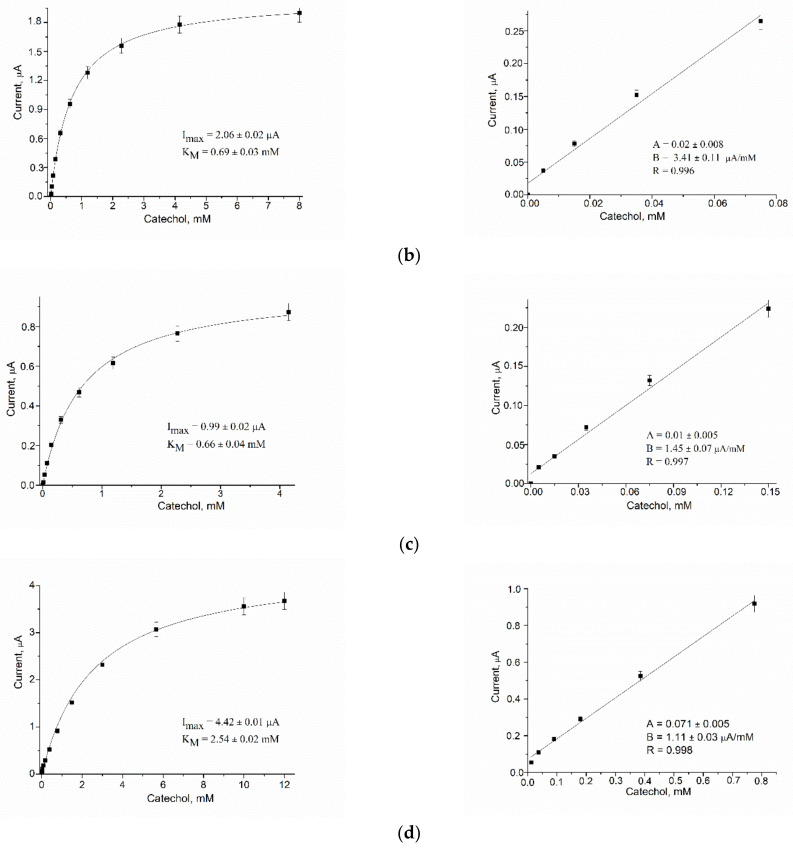
Calibration curves for catechol determination in wide (left) and linear (right) ranges using the laccase-based ABSs which contain electroactive NPs: (**a**) PtHCF, (**b**) AuHCF, (**c**) PtCeHCF, (**d**) AuCuHCF. Conditions: Ag/AgCl (reference electrode) in NaOAc buffer. Working potential: +230 mV.

We studied the effect of the most electroactive redox mediators on the analytical properties of the ABSs. The amperometric responses of different electrodes to the added substrate (catechol) were compared using CV (Figure 3) and chronoamperometry (Figure A1 and Figure A2). The calibration was performed by the stepwise addition of a standard analyte solution. Following the chronoamperograms, calibration curves for catechol determination in wide and linear ranges were plotted (Figure 4, Figure A2 and Figure A3).

Evaluation of the dependence of the operating characteristics of the ABSs was carried out according to the main parameters: a value of the ABS response when saturated with the substrate (I_max_), a value of the apparent Michaelis–Menten constant (K_M_^app^), the limit of detection (LOD), linearity, and sensitivity to catechol. Values for Imax and K_M_^app^ were calculated automatically and placed in the wide-range calibration curve field (Figure 4, Figure A2 and Figure A3). The sensitivity was calculated by taking into account the slope B from the linear regression graph (Figure 4, Figure A2 and Figure A3) and the square of the active electrode surface (7.3 mm^2^). Sensitivity data are expressed in standard SI units, A·M^−1^·m^−2^. This parameter characterizes the specific activity of the ABS, and in contrast to I_max_, it does not depend on the square of the electrode surface. The LOD was determined as a ratio of the triplicated standard deviation value of the blank to the slope “B”.

The analytical properties of the developed ABSs, as deduced from the graphs in Figure 4 (for ABSs with higher sensitivities) and in Figure A2 and Figure A3 (for ABSs with lower sensitivities), are summarized in Table 1. 

It was demonstrated that all the studied laccase- and NP-based ABSs exhibited significantly improved analytical parameters compared to the control ABS without NPs (Table 1). For example, ABSs based on CuCo, PdHCF, NiPtPd, and AgHCF showed an increase in sensitivity of 51, 13, 11, and 10-fold, respectively. 

Among the NPs which were studied in the current work, the most promising redox mediator was CuCo, which when coupled with laccase on the GE provided the highest sensitivity (4523 A M^−1^·m^−2^) and the lowest K_M_^app^ value (0.14 mM) for the ABS. This ABS may be useful for the analysis of catechol and other phenolic derivatives in real samples of drinking water, wastewater, food, and pharmaceuticals. 

#### 3.3.2. Morphologic Characterization of NPs 

**Figure A4:**
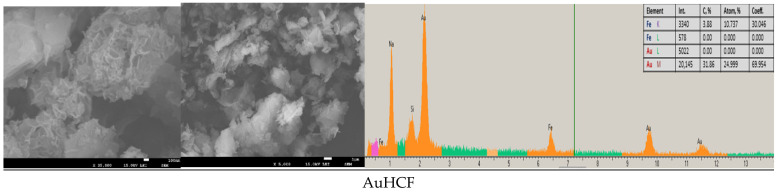

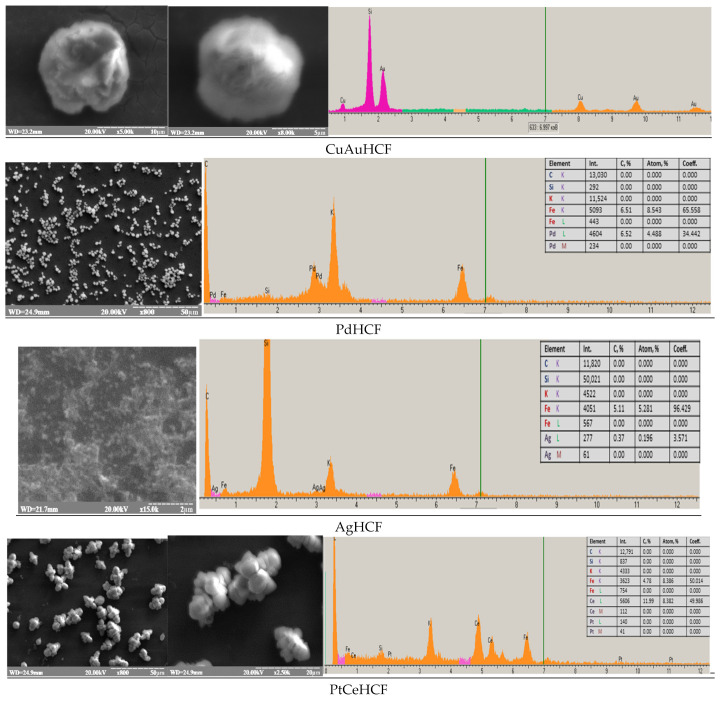
Structural and morphological characterizations of the synthesized NPs. The SEM (**left column**) with XRM **(right column**) images of NPs.

Some of the most effective NPs, containing hexacyanoferates (HCFs) of noble and transition metals, were characterized using SEM with X-ray spectral microanalysis (SEM-XRM). Figure A4 presents the SEM images which provided information on the size, distribution, and shape of the chemically synthesized NPs being studied. The XRM images show the characteristic peaks for the transition metals used for their synthesis. Morphologic analysis of other NPs which were used here for the development of ABSs was presented in our previous works [44,45,46,47]. 

It was demonstrated (data not shown) that the sizes of all studied materials did not satisfy the nanoscale criterion (less than 100 nm in all three dimensions). In some cases, they were nanoscale only in one dimension. Most likely, this is due to the aggregation of the initially formed NPs. To take into account this factor, we identified as NPs those materials whose nanoscale was confirmed by physical methods for at least one dimension.

### 3.4. Properties of the Most Effective Laccase/CuCo-Based ABS

#### 3.4.1. Optimization of Catechol Sensing

**Figure A5:**
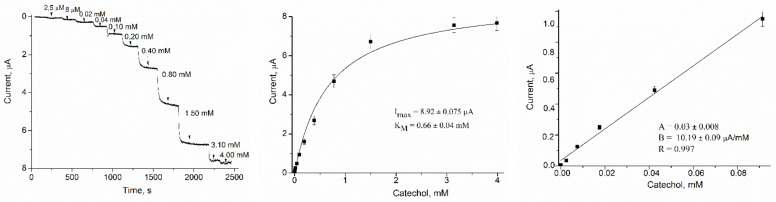
Chronoamperogram (**left**) and calibration curves for catechol determination in wide range (**middle**) and linear range (**right**) for the laccase/CuCo/GE, which contains 500 m-units of laccase and 1 µg of NPs. Conditions: working potential +230 mV vs. Ag/AgCl, NaOAc buffer, 23 °C.

To improve the effectiveness of catechol sensing, optimization of the ABS construction was carried out. For this purpose, the optimal quantities of laccase and CuCoNPs placed on the surface of the GE were determined. The analytical properties for one of the developed ABSs were deduced from the graphs in Figure A5 (for example). For other ABSs, the detailed descriptions are omitted here, namely chronoamperograms and calibration graphs with amperometric characteristics. The results are summarized in Table 2. 

Based on the data presented in Table 2, the highest sensitivity was achieved with 1 unit of laccase and 5 µg of CuCo on the GE surface (ABS-6). The resulting sensitivity was 42-fold higher in comparison with the control GE without NPs (ABS-10). However, the linearity range of the catechol assay for ABS-6 was rather narrow (up to 40 µM). Additionally, the sensing layer in ABS-6 was shown to be not stable enough because of large amounts of enzyme and NPs which were fixed with Nafion on the surface of GE. To achieve a compromise in analytical parameters, providing a rather high linearity, a high sensitivity, and a satisfactory stability of ABS, it was necessary to modify the GE with 1 µg CuCo and 100 m-units laccase. The developed sensor, ABS-2, was more stable and cost-effective, due to the small quantities of enzyme and NPs in the sensing layer. ABS-2 possessed a wide linear range (up to 18 mM) and rather high sensitivity (721 A·M^−1^ m^−2^), which was 7-fold higher than the control ABS-10.

#### 3.4.2. Selectivity 

**Figure 5:**
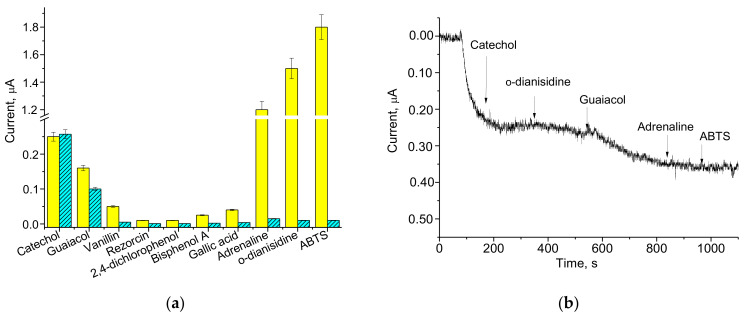
The selectivity tests for laccase/CuCo/GEs, which were covered with Nafion (**a**, yellow) or with DM (**a**, cyan; **b**): the current responses on the added analytes up to 0.2 mM concentration in the individual solutions (**a**) and in the mixture of sequentially added compounds (**b**).

Selectivity of the ABS towards the target analyte is of great importance, especially for the analysis of real samples. It is known that fungal laccases have very broad specificity, being able to oxidase many organic substrates, although with different effectiveness. In this paper, laccase/CuCo/GE was tested for its ability to respond to a number of organic substrates of laccase (Figure 5). It is worth mentioning that CuCo/GE did not react with all tested analytes. 

Two options of bioelectrodes laccase/CuCo/GEs were tested. The first electrode (#1) was covered with Nafion (see Section 2.4) and the second electrode (#2) was covered with DM (see Section 2.5). Figure 5a illustrates the results of the tests, which were performed for electrodes #1 and #2 with the individual natural substrates of laccase; Figure 5b demonstrates the chronoamperograms for electrode #2 as outputs on the mixture of the tested analytes. 

As shown in Figure 5a, the current responses for electrodes #1 (yellow) and #2 (cyan) on catechol are similar, while the response on guaiacol has some differences. At the same time, the outputs of both electrodes on adrenaline, o-dianisidine, and ABTS differ drastically: electrode #2 does not demonstrate any signals on the last analytes; perhaps they do not react with laccase. This phenomenon may be explained by the limited diffusion of rather large-sized analytes (in comparison with catechol and guaiacol) through the small pores of DM. Another reason for decreased penetration of adrenaline, o-dianisidine, and ABTS to enzyme in electrode #2 may be related to sorption/adhesion of these compounds on cellulose DM. Thus, the proposed ABS in option #2 is rather selective to catechol, especially in the mixture with non-phenolic substrates (see Figure 5b). 

### 3.5. Application of the Laccase/CuCo-Based ABS for Catechol Assay in the Model and Real Samples

To demonstrate the applicability of the developed ABS for determination of catechol, the model solution and the real sample were tested using the graphical calibration method in a SAT variant (see Section 2.6).

**Figure 6:**
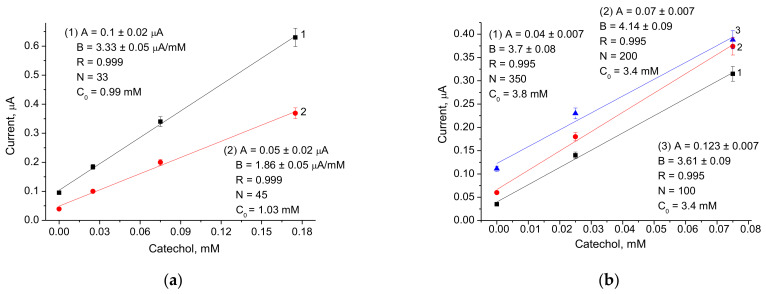
Assay of catechol using the laccase/CuCo-based ABS in the samples of wastewater supplemented with 1 mM catechol (**a**) and extract of green tea (**b**).

**Figure A6:**
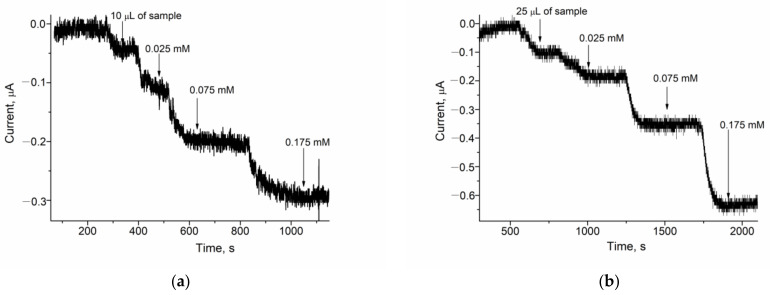
Determination of catechol content in the tested samples using the laccase/CuCo-based ABS. Chronoamperograms of the current responses upon catechol addition to the samples with dilutions: 33-fold (**a**) and 45-fold (**b**). The tested sample was wastewater supplemented with 1 mM catechol. Conditions: working potential +230 mV vs. Ag/AgCl, NaOAc buffer, 23 °C.

The sample of the model solution contained wastewater and added catechol up to 1 mM. The results of the analysis of the catechol content in this sample are presented in Figure A6 and Figure 6a. According to the obtained data (Figure 6a), the average concentration of catechol in the tested sample was 1.01 mM. Compared with the declared concentration of catechol added in the wastewater (1 mM), the difference between declared and estimated results (0.01 mM) was only 1%.

The results of green tea extract testing are presented in Figure 6a. The values of catechol that were determined in the samples with different dilutions correlated satisfactorily. The differences between the individual results (3.8, 3.4, and 3.4 mM) and the average value of catechol (3.55 mM) were less than 10%. The estimated average content of catechol in tea extract corresponds to 390 mg/L or 7.8 mg/g of dry leaves. Such catechol content in tea extract is similar to the reported data [34]. 

Thus, we have demonstrated the applicability of the developed ABS for catechol assay in the samples of wastewater and green tea.

## 4. Discussion

In our study, a number of catechol-sensitive ABSs based on laccase and redox nanomediators were constructed, and their analytical properties were characterized. It was demonstrated that all the studied NP-based ABSs had significantly improved analytical parameters compared to the control ABS without NPs. For example, the most effective ABS, containing the redox mediator CuCo, showed a 51-fold increase in sensitivity in comparison with the control ABS. This is significantly higher than the sensitivity reported earlier for laccase-based ABSs [38,49,50], except for our recent work [19], where we described the electroactive CuCeNPs coupled with porous gold as a platform for the construction of oxidase-based ABSs. We have demonstrated here the possibility to improve the selectivity of ABS to catechol in the mixture with several non-phenolic substrates of laccase. For this purpose, particularly for fixing laccase/CuCo on the surface of electrode, a cellulose dialysis membrane was used.

The question is raised regarding the reason for such effectiveness in electroactive NPs: Is the redox activity of their mediators the sole advantage that causes significantly enhanced sensitivity in laccase-based ABSs?

It is worth mentioning that the electroactive NPs described in our work are artificial peroxidases (POs) or PO-like nanozymes. It was demonstrated by us earlier [19,24,44,45,46,47,48], and in this work (data not shown) that all NPs used here possess pseudo-PO activity that makes them able to decompose H_2_O_2_ both in solution and on amperometric electrodes. 

To date, most of the pathway mechanisms in Cu-promoted laccase activity are still unknown [1,2,3]. The traditional understanding of the mechanisms of laccase catalytic activity was that laccase does not require H_2_O_2_ as a co-substrate or an additional cofactor for an enzymatic reaction, nor does laccase produce this compound [51]. However, that postulate was disproven in past decades, when an alternative mechanism of O_2_ reduction mediated with laccase was discovered [1,2,7,8,52,53,54,55,56,57,58].

Many microbial producers used for LMCO isolation have been described to date. A number of recombinant and mutant strains have been proposed [1,2,3,4,5,9,14] to improve the properties of this enzyme which plays important roles in many fields of science, biotechnology, industry, and medicine. Various properties of LMCO were studied, including substrate specificity, structure, and different mechanisms of catalytic activity [1,2,3,7,52,53,54,55,56,57,58]. 

The electron transfer (ET) process in laccases, and the mechanism of O_2_ reduction as elucidated through spectroscopic, kinetic, crystallographic, and computational data, have been described in several papers [1,2,3,52,53,54,55,56]. Laccases classed as LMCOs contain a minimum of four copper sites, designated as T1, T2, and binuclear T3. Each Cu site has unique spectroscopic features. According to the proposed mechanisms of electron transfer, the substrate is oxidized near T1, and ET occurs through the laccase via the Cys-His pathway to the T2/T3 trinuclear copper cluster. In the T2/T3 cluster water is produced from O_2_ due to the 4H^+^/4e^–^ reduction process. Thus, laccase catalyzes the coupling of the four single-electron oxidations in a variety of substrates with the four-electron reduction of O_2_ to water. 

The main approaches to investigating the ET mechanism are spectroscopic characterization of reaction intermediates, kinetic analysis, oxidation of substrates designed to serve as mechanistic probes, the effect of the addition of radical traps, X-ray devices, and computational analysis [2,3,7,53,54,55,56,57,58]. As the result of these high-throughput physical methods, formation of two sequential intermediates, namely “peroxide (PI)” and the “native (NI)”, were hypothesized. During the two reduction steps of this process, cleavage of the O-O bond in a PI effectively leads to the formation of an NI, which is then poised for rapid proton-coupled reduction to generate H_2_O and continue the catalytic cycle. Crystallographic study using a high-intensity X-ray synchrotron beam radiation approach has confirmed this hypothesis [57]. 

We demonstrated in our study that the presence of electroactive PO-like nanozymes in the sensing layer of the laccase-based ABS couses an improvement in analytical characteristics of the biosensor. We believe that our finding concerning the positive effect of the nano-sized PO mimetics on the significant enhancing sensitivity of the developed ABSs indirectly confirms the concept of the existence of the PI intermediate under laccase catalysis. Our results obtained by simple electrochemical methods make a small contribution to the elucidation of the mechanism of catalytic activity of laccase. A deeper knowledge of the pathways of Cu-promoted laccase activity—namely details of the changes in ET and/or proton transfer rates—will provide better understanding of how nature controls the process of O_2_ reduction to H_2_O [1,2,3,7,56,57,58,59]. Additionally, this knowledge will also be of practical importance for biomedical applications and industrial processes. 

## Figures and Tables

**Table 1:** The main operational parameters of the constructed ABSs for catechol analysis.

Composition of ABS	Sensitivity, A·M^−1^·m^−2^	Linear Range, up to, mM	LOD, µA	K_M_^app^, mM	I_max_, µA
Laccase/GE	89	0.30	1	0.70	0.65
Laccase/CuAuHCF/GE	152	0.80	2	2.54	4.42
Laccase/PtCeHCF/GE	199	0.14	1	0.66	0.99
Laccase/PtHCF/GE	517	0.15	0.1	0.49	2.41
Laccase/AuCo/GE	630	0.06	0.1	1.10	2.05
Laccase/AuHCF/GE	757	0.08	0.2	1.27	6.02
Laccase/AgHCF/GE	881	0.15	0.2	0.80	5.24
Laccase/NiPtPd/GE	990	0.08	0.1	2.69	5.76
Laccase/PdHCF/GE	1137	0.10	0.2	0.33	3.48
Laccase/CuCo/GE	4523	0.09	0.2	0.14	7.36
* Laccase/GE	103	1.60	4	2.57	3.13
* Laccase/gAu/GE	295	0.40	1	1.56	3.96
* Laccase/gFeHCF/GE	339	0.80	1	2.75	8.55
* Laccase/PtRu/GE	551	0.40	1	1.66	9.14
* Laccase/gCuHCF/GE	762	0.20	0.5	2.03	7.31

* Note: This bioelement was fixed on the surface of the GE with the cathodic polymer CP59.

**Table 2:** Analytical properties of the ABS as the dependence on the composition of the sensing layer.

ABS, No	Sensing Layer		Sensitivity, A·M^−1^ m^−2^	Linear Range, up to, mM	K_M_^app^_,_ mM	I_max,_ µM
	Laccase, m-Units	CuCo, µg				
1	50	1	220	0.16	2.48	2.94
2	100	1	721	0.18	1.05	5.18
3	200	1	750	0.09	1.49	6.37
4	500	1	1441	0.09	0.66	8.92
5	500	5	3435	0.04	0.59	7.57
6	1000	5	4495	0.04	0.25	7.48
7	200	2	770	0.10	1.67	7.70
8	200	4	820	0.18	1.80	8.17
9	200	10	188	0.76	5.27	8.62
10	100 (Control)	0	107	0.80	1.67	1.76

## Data Availability

The data are included within the present article.

## Appendix A

Appendix A presents amperometric characteristics of the developed laccase/NP-based ABSs as the current responses to catechol addition (Figure A1, Figure A2, Figure A3, Figure A4 and Figure A5), data of the structural and morphological characterizations of the synthesized NPs (Figure A4), and the example of catechol content assay in real samples using the laccase/CuCo-based ABS (Figure A6).

## References

[1] Jeon J.-R., Baldrian P., Murugesan K., Chang Y.-S. (2012). Minireview Laccase-catalysed oxidations of naturally occurring phenols: From in vivo biosynthetic pathways to green synthetic applications. Microb. Biotechnol..

[2] Reiss R., Ihssen J., Richter M., Eichhorn E., Schilling B., Thöny-Meyer L. (2013). Laccase versus laccase-like multi-copper oxidase: A comparative study of similar enzymes with diverse substrate spectra. PLoS ONE.

[3] Gulsunoglu-Konuskan Z., Kilic-Akyilmaz M. (2022). Microbial Bioconversion of Phenolic Compounds in Agro-industrial Wastes: A Review of Mechanisms and Effective Factors. J. Agric. Food Chem..

[4] Bhadra F., Gupta A., Vasundhara M., Reddy M.S. (2022). Endophytic fungi: A potential source of industrial enzyme producers. 3 Biotech..

[5] Mayolo-Deloisa K., González-González M., Rito-Palomares M. (2020). Laccases in Food Industry: Bioprocessing, Potential Industrial and Biotechnological Applications. Front. Bioeng. Biotechnol..

[6] Colella A., De Chiaro A., Lettera V. (2021). In Situ Wood Fiber Dyeing Through Laccase Catalysis for Fiberboard Production. Front. Bioeng. Biotechnol..

[7] Perna V., Meyer A.S., Holck J., Eltis L.D., Eijsink V.G.H., Agger J.W. (2020). Laccase-Catalyzed Oxidation of Lignin Induces Production of H_2_O_2_. ACS Sustain. Chem. Eng..

[8] Shin S.K., Hyeon J.E., Joo Y.-C., Jeong D.W., You S.K., Han S.O. (2019). Effective melanin degradation by a synergistic laccase-peroxidase enzyme complex for skin whitening and other practical applications. Int. J. Biol. Macromol..

[9] Martini M.C., Berini F., Ausec L., Casciello C., Vacca C., Pistorio M., Lagares A., Mandic-Mulec I., Marinelli F., Del Papa M.F. (2021). Identification and Characterization of a Novel Plasmid-Encoded Laccase-Like Multicopper Oxidase from Ochrobactrum sp. BF15 Isolated from an On-Farm Bio-Purification System. Food. Technol. Biotechnol..

[10] Global “Laccase Market” (2022–2028) Report. https://www.wicz.com/story/45797138/global-laccase.

[11] Sakurai T., Kataoka K. (2007). Basic and applied features of multicopper oxidases, CueO, bilirubin oxidase, and laccase. Chem Rec..

[12] Bilal M., Ashraf S.S., Cui J., Lou W.-Y., Franco M., Mulla S.I., Iqbal H.M.N. (2021). Harnessing the biocatalytic attributes and applied perspectives of nanoengineered laccases—A review. Int. J. Biol. Macromol..

[13] Kumar A., Singh D., Sharma K.K., Arora S., Singh A.K., Gill S.S., Singhal B. (2017). Gel-Based Purification and Biochemical Study of Laccase Isozymes from Ganoderma sp. and Its Role in Enhanced Cotton Callogenesis. Front. Microbiol..

[14] Chiadò A., Bosco F., Bardelli M., Simonelli L., Pedotti M., Marmo L., Varani L. (2021). Rational engineering of the lccβ T. versicolor laccase for the mediator-less oxidation of large polycyclic aromatic hydrocarbons. Comput. Struct. Biotechnol. J..

[15] Wang L., Ding X., Huang Q., Hu B., Liang L., Wang Q. (2022). Gllac7 Is Induced by Agricultural and Forestry Residues and Exhibits Allelic Expression Bias in Ganoderma lucidum. Front. Microbiol..

[16] Pezzella C., Guarino L., Piscitelli A. (2015). How to enjoy laccases. Cell. Mol. Life Sci..

[17] Yoshida H. (1883). Chemistry of lacquer (urushi) part I. J. Chem. Soc. Trans..

[18] Demkiv O.M., Gayda G.Z., Broda D., Gonchar M.V. (2021). Extracellular laccase from Monilinia fructicola: Isolation, primary characterization and application. Cell Biol. Int..

[19] Stasyuk N., Demkiv O., Gayda G., Zakalskiy A., Klepach H., Bisko N., Gonchar M., Nisnevitch M. (2022). Highly Porous 3D Gold Enhances Sensitivity of Amperometric Biosensors Based on Oxidases and CuCe Nanoparticles. Biosensors.

[20] Miyata M., Kitazumi Y., Shirai O., Kataoka K., Kano K. (2020). Diffusion-limited biosensing of dissolved oxygen by direct electron transfer-type bioelectrocatalysis of multi-copper oxidases immobilized on porous gold microelectrodes. J. Electroanal. Chem..

[21] Dinu A., Apetrei C. (2022). Quantification of Tyrosine in Pharmaceuticals with the New Biosensor Based on Laccase-Modified Polypyrrole Polymeric Thin Film. Polymers..

[22] Chen H., Simoska O., Lim K., Grattieri M., Yuan M., Dong F., Lee Y.S., Beaver K., Weliwatte S., Gaffney E.M. (2020). Fundamentals, applications, and future directions of bioelectrocatalysis. Chem. Rev..

[23] Kadam A.A., Saratale G.D., Ghodake G.S., Saratale R.G., Shahzad A., Magotra V.K., Kumar M., Palem R.R., Sung J.-S. (2022). Recent Advances in the Development of Laccase-Based Biosensors via Nano-Immobilization Techniques. Chemosensors.

[24] Demkiv O., Stasyuk N., Serkiz R., Gayda G., Nisnevitch M., Gonchar M. (2021). Peroxidase-Like Metal-Based Nanozymes: Synthesis, Catalytic Properties, and Analytical Application. Appl. Sci..

[25] Flickinger C.W. (1976). The benzenediols: Catechol, resorcinol and hydroquinone—A review of the industrial toxicology and current industrial exposure limits. Am. Ind. Hyg. Assoc. J..

[26] Lee B.P., Birkedal H., Lee H. (2019). Editorial: Catechol and Polyphenol Chemistry for Smart Polymers. Front. Chem..

[27] Nsanzamahoro S., Mutuyimana F.P., Han Y., Ma S., Na M., Liu J., Ma Y., Ren C., Chen H., Chen X. (2018). Highly selective and sensitive detection of catechol by one step synthesized highly fluorescent and water-soluble silicon nanoparticles. Sens. Actuat. B Chem..

[28] Jabbari S., Dabirmanesh B., Khajeh K. (2015). Specificity enhancement towards phenolic substrate by immobilization of laccase on surface plasmon resonance sensor chip. J. Mol. Catal. B Enzym..

[29] Wang Y., Li Y., Bao X., Han J., Xia J., Tian X., Ni L. (2016). A smartphone-based colorimetric reader coupled with a remote server for rapid on-site catechols analysis. Talanta.

[30] Castrovilli M.C., Bolognesi P., Chiarinelli J., Avaldi L., Cartoni A., Calandra P., Tempesta E., Giardi M.T., Antonacci A., Arduini F. (2020). Electrospray deposition as a smart technique for laccase immobilisation on carbon black-nanomodified screen-printed electrodes. Biosens. Bioelectron..

[31] Li Z., Zeng H.-Z., Cao X.-J., Li H.-B., Long Y.-W., Feng B., Lv S.-B. (2021). High-sensitive sensor for the simultaneous determination of phenolics based on multi-walled carbon nanotube/NiCoAl hydrotalcite electrode material. Microchim. Acta.

[32] Pillai R., Preetha S., Narasimhamurthy B., Lekshmi I.C. (2022). Biosensing of catechol via amperometry using laccase immobilized nickel oxide/graphite modified screen-printed electrodes. Mater. Today: Proc..

[33] Melak F., Redi M., Tessema M., Alemayehu E. (2013). Electrochemical determination of catechol in tea samples using anthraquinone modified carbon paste electrode. Nat. Sci..

[34] Broli N., Vallja L., Shehu A., Vasjar M. (2018). Determination of catechol in extract of tea using carbon paste electrode modified with banana tissue. J. Food Process Preserv..

[35] Nellaiappan S., Kumar S. (2018). Selective amperometric and flow injection analysis of 1,2-dihydroxy benzene isomer in presence of 1,3- and 1,4-dihydroxy benzene isomers using palladium nanoparticles-chitosan modified ITO electrode. Sens. Actuat. B Chem..

[36] Zhou Y., Tang L., Zeng G., Chen J., Cai Y., Zhang Y., Yang G., Liu Y., Zhang C., Tang W. (2014). Mesoporous carbon nitride based biosensor for highly sensitive and selective analysis of phenol and catechol in compost bioremediation. Biosens. Bioelectron..

[37] Rodrigues R.C., Ortiz C., Berenguer-Murcia Á., Torres R., Fernández-Lafuente R. (2013). Modifying enzyme activity and selectivity by immobilization. Chem. Soc. Rev..

[38] Deniz S.A., Goker S., Toppare L., Soylemez S. (2022). Fabrication of D–A–D type conducting polymer, carbon nanotubes and silica nanoparticle-based laccase biosensor for catechol detection. New J. Chem..

[39] Qi H., Zhang C. (2005). Simultaneous determination of hydroquinone and catechol at a glassy carbon electrode modified with multiwall carbon nanotubes. Electroanalysis.

[40] Hammani H., Laghrib F., Farahi A., Lahrich S., El Achaby M., El Harfi K., Aboulkas A., Bakasse M., El Mhammedi M.A. (2018). Date stone based activated carbon/graphite electrode for catechol analysis: Physico-chemical properties and application in beverage samples. New J. Chem..

[41] Arya Nair J.S., Saisree S., Sandhya K.Y. (2022). Picomolar level electrochemical detection of hydroquinone, catechol and resorcinol simultaneously using a MoS2 nano-flower decorated graphene. Analyst.

[42] Harisha B.K., Kumara Swamy V.E., Ebenso E.E. (2018). Poly (glycine) modified carbon paste electrode for simultaneous determination of catechol and hydroquinone: A voltammetric study. J. Electroanal. Chem..

[43] Liu H., Zhou P., Wu X., Sun J., Chen S. (2015). Radical Scavenging by Acetone: A New Perspective to Understand Laccase/ABTS Inactivation and to Recover Redox Mediator. Molecules.

[44] Gayda G.Z., Demkiv O.M., Gurianov Y., Serkiz R.Y., Klepach H.M., Gonchar M.V., Nisnevitch M. (2021). “Green” Prussian Blue Analogues as Peroxidase Mimetics for Amperometric Sensing and Biosensing. Biosensors.

[45] Gayda G.Z., Demkiv O.M., Stasyuk N.Y., Serkiz R.Y., Gonchar M.V., Nisnevitch M. (2019). Metallic nanoparticles obtained via “green” synthesis as a platform for biosensor construction. Appl. Sci..

[46] Stasyuk N., Gayda G., Zakalskiy A., Zakalska O., Serkiz R., Gonchar M. (2019). Amperometric biosensors based on oxidases and PtRu nanoparticles as artificial peroxidase. Food Chem..

[47] Stasyuk N., Gayda G., Demkiv O., Darmohray L., Gonchar M., Nisnevitch M. (2021). Amperometric biosensors for L-arginine determination based on L-arginine oxidase and peroxidase-like nanozymes. Appl. Sci..

[48] Stasyuk N., Demkiv O., Gayda G., Zakalska O., Zakalskiy A., Serkiz R., Kavetskyy T., Gonchar M. (2022). Reusable alcohol oxidase-nPtCu/alginate beads for highly sensitive ethanol assay in beverages. RSC Adv..

[49] Othman A.M., Wollenberger U. (2020). Amperometric biosensor based on coupling aminated laccase to functionalized carbon nanotubes for phenolics detection. Int. J. Biol. Macromol..

[50] Rodríguez-Delgado M.M., Alemán-Nava G.S., Rodríguez-Delgado J.M., Dieck-Assad G., Martínez-Chapa S.O., Barceló D., Parra R. (2015). Laccase-based biosensors for detection of phenolic compounds. TrAC Trends Anal. Chem..

[51] Thurston C.F. (1994). The structure and function of fungal laccases. Microbiology.

[52] Schlosser D., Höfer C. (2002). Laccase-Catalyzed Oxidation of Mn in the Presence of Natural Mn Chelators as a Novel Source of Extracellular H_2_O_2_ Production and Its Impact on Manganese Peroxidase. Appl. Environ. Microbiol..

[53] Ming C., Lin W., Tian T., Xue-Cai L., Zai Z., Ruo-Chun Y., Ji-Hu S., Jiang-Feng D. (2017). Radical Mechanism of Laccase-Catalyzed Catechol Ring-Opening. Acta Phys. Chim. Sin..

[54] Jones S.M., Solomon E.I. (2015). Electron transfer and reaction mechanism of laccases. Cell. Mol. Life Sci..

[55] Clément R., Wang X., Biaso F., Ilbert M., Mazurenko I., Lojou E. (2021). Mutations in the coordination spheres of T1 Cu affect Cu^2+^-activation of the laccase from Thermus thermophilus. Biochimie.

[56] Mazurenko I., Adachi T., Ezraty B., Ilbert M., Sowa K., Lojou E. (2022). Electrochemistry of copper efflux oxidase-like multicopper oxidases involved in copper homeostasis. Curr. Opin. Electrochem..

[57] Ferraroni M., Myasoedova N.M., Schmatchenko V., Leontievsky A.A., Golovleva L.A., Scozzafava A., Briganti F. (2007). Crystal structure of a blue laccase from Lentinus tigrinus: Evidences for intermediates in the molecular oxygen reductive splitting by multicopper oxidases. BMC Struct. Biol..

[58] Davydov R., Herzog A.E., Jodts R.J., Karlin K.D., Hoffman B.M. (2022). End-On Copper(I) Superoxo and Cu(II) Peroxo and Hydroperoxo Complexes Generated by Cryoreduction/Annealing and Characterized by EPR/ENDOR Spectroscopy. J. Am. Chem. Soc..

[59] Giardina P., Faraco V., Pezzella C., Piscitelli A., Vanhulle S., Sannia G. (2010). Laccases: A never-ending story. Cell. Mol. Life Sci..

